# Effects of Distiller’s Grains Biochar and *Lactobacillus plantarum* on the Remediation of Cd-Pb-Zn-Contaminated Soil and Growth of Sorghum-Sudangrass

**DOI:** 10.3390/microorganisms12122592

**Published:** 2024-12-14

**Authors:** Guangxu Zhu, Yufeng Li, Dandan Cheng, Rongkun Chen, Yunyan Wang, Qiang Tu

**Affiliations:** 1 College of Biology and Environment Engineering, Guiyang University, Guiyang 550005, China; 2 Helmholtz International Lab for Anti-Infectives, Shandong University–Helmholtz Institute of Biotechnology, State Key Laboratory of Microbial Technology, Shandong University, Qingdao 266237, China; liwoyuan200011@163.com; 3 Institute of Synthetic Biology Industry, Hunan University of Arts and Science, Changde 415000, China

**Keywords:** heavy metals, soil pollution, combined remediation, distiller’s grains biochar, *Lactobacillus plantarum*

## Abstract

Soil contamination with heavy metals is a significant environmental issue that adversely affects plant growth and agricultural productivity. Biochar and microbial inoculants have emerged as a promising approach to solving this problem, and previous studies have focused more on the remediation effects of single types of materials on heavy metal soil pollution. This study examined the impact of both standalone and combined applications of distiller’s grains biochar, *Lactobacillus plantarum* thallus, and the bacterial supernatant on the availability of cadmium (Cd), lead (Pb), and zinc (Zn) in soil, its physicochemical features, and its enzyme activities; this study also examined the growth, physiological and biochemical characteristics, and heavy metal accumulation of Sorghum-sudangrass. The findings suggest that the application of distiller’s grains biochar, *Lactobacillus plantarum* thallus, and the bacterial supernatant can improve the soil’s physical and chemical properties and enhance soil enzyme activity while reducing the availability of heavy metals in the soil. Furthermore, the addition of these materials promoted plant growth, increased stress resistance, and significantly decreased the accumulation of heavy metals in the plants. A thorough analysis of the results shows that applying 0.025% *Lactobacillus plantarum* thallus along with 4.4% distiller’s grains biochar produced the best results.

## 1. Introduction

Recent years have seen a significant increase in heavy metal soil pollution due to the rapid advancement of urbanization and industrialization, along with the improper use of pesticides and fertilizers in agricultural practices [1,2]. This type of pollution is typically a long-term accumulation process, characterized by prolonged residual times, strong concealment, and high toxicity [3]. The heavy metals present in the soil can diminish soil fertility, adversely affect crop growth, reduce yields, and enter the human body through the food chain, posing serious health risks [4,5]. Consequently, mitigating heavy metal soil contamination is essential for ensuring food security; nevertheless, managing this pollution has emerged as a significant and complex area of research.

Generally, there are two types of remediation strategies for heavy metal soil pollution: one focuses on extracting heavy metal elements from the soil to lower their concentrations, while the other aims to reduce the mobility and bioavailability of heavy metal ions in the soil to limit their incorporation into the food chain [6,7]. Recent advancements in heavy metal soil pollution remediation technologies have led to the common use of physical, chemical, and biological remediation techniques [8].

Physical remediation methods include soil capping, microwave thermal remediation, and electrokinetic remediation. Although these methods are effective and provide quick results, they often require substantial engineering efforts, are time-consuming and labor-intensive, and yield low economic benefits, making them unsuitable for large-scale applications [9]. Chemical remediation methods encompass leaching and in situ chemical stabilization. However, the improper use of chemical leachants can lead to deeper pollution in the soil environment. In situ chemical stabilization technology is favored for its notable effectiveness, rapid reaction speed, relative ease of operation, and suitability for large-area pollution management, making it widely regarded as the most appropriate remediation technology for moderately contaminated farmland [10,11]. Biological remediation techniques generally refer to the use of plants, animals, and microorganisms to remediate heavy metals in the soil [12,13]. One of these methods is microbial remediation, which uses native or foreign microorganisms to block the movement and availability of heavy metals by complexing, chelating, and precipitating them [14]. Microbial remediation technology offers advantages such as being environmentally friendly, low-cost, and capable of the in situ remediation of contaminated soils [15,16,17]. However, whether physical, chemical, or biological remediation methods are employed, each approach has its limitations. The key challenge lies in effectively combining two or more remediation methods to create a synergistic effect, thereby enhancing remediation efficiency while minimizing the potential hazards associated with the remediation process.

While microbial remediation offers numerous benefits, the natural growth and reproduction of introduced microorganisms in environmental settings are subject to various limiting factors [18]. Consequently, selecting appropriate microbial strains is essential [19]. Moreover, supplementing external nutrients such as carbon and nitrogen sources can boost the proliferation of microorganisms and, to some extent, improve their remediation efficiency [20]. Therefore, fully utilizing microbial resources in conjunction with environmentally friendly materials for the composite remediation of moderately contaminated farmland presents a sustainable and eco-friendly approach.

In recent years, biochar has gained significant attention for its widespread application in the remediation of heavy-metal-contaminated soils [21]. The pyrolysis of biomass under high temperatures and low oxygen conditions produces biochar. The common feedstocks used to produce biochar include crop residues, plant leaves and branches, sludge, and animal manure [22,23]. The cheap, easy-to-find, and many functional groups on the surface of biochar make it a good material for changing heavy metals from their free state to a stable state through adsorption, precipitation, and ion exchange reactions. [24,25,26]. Consequently, heavy-metal-contaminated soil frequently utilizes biochar as a treatment agent. As an environmentally friendly material, biochar not only effectively adsorbs heavy metals in the soil but also enhances the soil’s nutrient retention and supply capacity, improves soil conditions, and provides nutrients and a habitat for microbial growth and reproduction [27,28]. By working together, microorganisms and biochar can stabilize heavy metals in the soil. This makes it possible to reuse agricultural waste and provides a new way to clean up heavy-metal-contaminated soils. Lactic acid bacteria (LAB) are a group of Gram-positive, non-spore-forming, anaerobic bacteria that include several genera such as *Lactobacillus, Pediococcus, Leuconostoc, Lactococcus, Streptococcus*, and *Bifidobacterium* [29]. Numerous studies have demonstrated that certain strains of lactic acid bacteria exhibit notable tolerance and adsorption capabilities toward heavy metals [30,31,32]. The adsorption of heavy metal ions by lactic acid bacteria primarily relies on the surface adsorption mechanism, whereby free, non-metabolized heavy metal ions bind to the cell wall through electrostatic interactions between positive and negative charges. Teichoic acids, peptidoglycan, and various neutral polysaccharides primarily compose the cell wall, forming a network structure capable of adsorbing and adhering to heavy metals. The proteins on the surface of lactic acid bacteria possess numerous negatively charged functional groups, which effectively bind to metal cations [33]. Additionally, lactic acid, a metabolic byproduct, contributes to the creation of an acidic environment that enhances the solubility of heavy metal complexes. Large biomolecules like polysaccharides, nucleic acids, and peptides are made when lactic acid bacteria break down food. They have groups on their surfaces called HPO_4_^2^⁻, OH⁻, and COO⁻ that can interact with metal ions through electrostatic adsorption [34].

Chinese liquor is a vital industry in Guizhou Province. In recent years, the liquor industry in Guizhou Province has experienced substantial growth, with the production of distiller grains increasing annually. Distiller’s grains contain a rich array of nutrients, such as proteins, fats, cellulose, and hemicellulose, which confer significant potential for development and utilization [35]. However, their high moisture content and acidity present major challenges for their reuse. Effectively and sustainably utilizing distillers’ grains is essential for maximizing resource efficiency within the liquor industry. Currently, Guizhou Province treats the majority of distiller’s grains as waste, often using simple and rudimentary methods like stacking, landfilling, or incineration. Such practices not only lead to resource wastage but also pose considerable risks, including environmental pollution. Therefore, actively pursuing research on the reuse of distiller’s grains can significantly mitigate resource waste and environmental issues while generating new ecological and economic value.

In this current study, we conducted potted experiments using Sorghum-sudangrass as the test plant. The goal of this study was to make biochar from distiller’s grains. The experimental strains used were *Lactobacillus plantarum*, which we isolated during our earlier work on making garbage enzymes. These strains have demonstrated strong tolerance and adsorption capabilities toward heavy metals. This research will investigate the remediation of heavy-metal-contaminated soils in mining areas using distiller’s grains biochar and *Lactobacillus plantarum*. The findings are anticipated to provide theoretical foundations and practical insights for the remediation of contaminated farmland and the promotion of safe agricultural production in the future.

## 2. Materials and Methods

### 2.1. Materials Preparation

The distiller’s grains used in this experiment were sourced from a distillery in Maotai Town, Renhuai City, Guizhou Province. After removing large impurities, they were air-dried, transferred to an oven, and dried at a temperature of approximately 50 °C to 70 °C until reaching a constant weight. The distiller’s grains were ground and passed through a 100-mesh sieve, packed into aluminum containers, compressed to eliminate voids, covered, and tightly wrapped in aluminum foil. Subsequently, the distiller’s grains were placed in a tube furnace and heated under limited oxygen conditions at a rate of 10 °C per minute until it reached 450 °C. Once the target temperature was achieved, it was maintained for 2 h, then allowed to cool to room temperature before being removed, sieved again through a 100-mesh sieve, and sealed in bags.

The bacterial strain selected for this study was a high-abundance *Lactobacillus plantarum*, which was isolated and identified from a self-made garbage enzyme in preliminary experiments. The isolated *Lactobacillus plantarum* strain was inoculated onto MRS solid culture (Solarbio, Beijing, China) medium and incubated at 35 °C for 48 h. A total of 49.3 g of MRS broth was measured, distilled water was added to a final volume of 1000 mL, and the pH was adjusted to approximately 6.5. The medium was then autoclaved at high pressure for 20 min to prepare the liquid culture medium. Single colonies from the MRS solid medium were picked and inoculated into the MRS broth. This culture was incubated at 35 °C for 48 h. After washing twice with sterile water, a bacterial suspension with an approximate concentration of 40 × 10^9^ cfu·mL^−1^ was prepared. The bacterial suspension was centrifuged at 5000 r·min^−1^, and the supernatant was collected. The bacterial thallus was then diluted with 0.85% saline and centrifuged again, and this process was repeated three times to remove impurities from the culture medium. The final bacterial thallus was resuspended in 0.85% saline and stored for future use. Additionally, the homogenized bacterial suspension was centrifuged and subsequently freeze-dried to prepare lyophilized bacterial thallus.

The test soil was collected from farmland surrounding a typical karst mining area in northwestern Guizhou Province. The soil is yellow-brown soil with a pH of 7.59, a total organic matter content of 39.76 g/kg, and a cation exchange capacity (CEC) of 27.47 cmol/kg. The mass fractions of sand, silt, and clay are 6.8%, 19.5%, and 73.7%, respectively. The total concentrations of Cd, Pb, and Zn in the soil were measured at 3.65 mg·kg^−1^, 870.50 mg·kg^−1^, and 1996.25 mg·kg^−1^, respectively, which correspond to 5.54, 19.34, and 24.23 times the background values for Guizhou Province. The test plant used in this study was Sorghum-sudangrass, a genus of annual herbaceous plants belonging to the Poaceae family that is characterized by a high yield, rapid growth, and strong adaptability, making it an excellent forage crop variety. The seeds of Sorghum-sudangrass were purchased from a local seed market in Guiyang City, Guizhou Province.

### 2.2. Experimental Setting

A total of 9 treatments were established, with each treatment replicated 4 times, as detailed in Table 1. Plastic pots with a diameter of 20 cm and a height of 23 cm were used, with each pot containing 3.6 kg of contaminated soil. Prior to planting the seeds, the corresponding remediation materials were added to the pots. After a 14-day stabilization period, the seeds were sown. Depending on the germination of the plants, some were selectively removed, leaving 5 to 7 plants per pot. To maintain optimal growth conditions, periodic watering was carried out to keep the soil moisture content at approximately 60% of the field capacity.

### 2.3. Samples Collection and Physicochemical Analysis

The samples were collected from both the above-ground and root parts after two months of plant maturation, carefully labeled, and analyzed for various indicators. The plants were rinsed with tap water, followed by three washes with deionized water. Subsequently, the samples were freeze-dried, ground, and preserved for further analysis. The contents of Cd, Pb, and Zn in both the aerial and root tissues of Sorghum-sudangrass were determined using a HNO_3_-HClO_4_ mixed acid digestion method [36]. The determination of the chlorophyll, soluble protein, proline (Pro) contents and superoxide dismutase (SOD) activity in the plants was performed using the ethanol extraction method, Coomassie Brilliant Blue G-250 method, acidic ninhydrin method, and nitroblue tetrazolium photochemical reduction method, respectively. The contents of malondialdehyde (MDA) and soluble sugars were determined using the thiobarbituric acid method [37].

The surface layer of rhizosphere soil (0–5 cm depth) was evenly removed from each treatment pot, with 100 g of soil collected for each treatment. The soil was then allowed to airdry naturally and subsequently passed through a 100-mesh sieve after grinding. The soil’ sucrose enzyme activity was measured using the Na_2_S_2_O_3_ titration method, the urease activity was determined using the phenol–sodium hypochlorite colorimetric method, the catalase activity was assessed using the potassium permanganate titration method, and the alkaline phosphatase activity was measured using the disodium phenyl phosphate colorimetric method [38,39]. Additionally, the pH of the soil suspension (1:5, *w*/*v*) was measured using a pH meter (PHS-3E, Leici, Shanghai, China). The soil’s organic matter and total nitrogen contents were determined using an elemental analyzer (vario MACRO cube, Elementar, Frankfurt, Germany). The total phosphorus and total potassium contents were measured using the molybdenum blue colorimetric method and atomic absorption spectrophotometry, respectively. The alkaline nitrogen content was measured using the alkaline hydrolysis diffusion method, the available phosphorus content was determined using the molybdenum–antimony colorimetric method, and the available potassium was measured using the neutral ammonium acetate extraction–flame photometry method [40]. The extraction of available Cd, Pb, and Zn in the soil was performed using the DTPA-TEA-CaCl_2_ extraction method [41].

The soil’s enzyme activity and the physio-biochemical indicators in the test plant were measured using a UV–visible spectrophotometer (UV-5100, Yuanxi, Shanghai, China). The metal concentrations in the digestive and extraction solutions were determined by inductively coupled plasma optical emission spectrometry (ICP-OES, Optima 5300DV, PerkinElmer, Waltham, MA, USA). Certified reference materials (GSS- and GSV-) obtained from the Center of National Standard Reference Materials of China, as well as blank samples [42], were included in each batch of analyses for quality assurance procedures. Good agreement was obtained between our method and certified values. All samples were analyzed in duplicate and the analytical precision was accepted when the relative standard deviation was within 5%.

### 2.4. Statistical Analysis

The data were subjected to statistical analysis using SPSS 22.0 (Chicago, IL, USA), and graphs were generated using Origin 2022 (Austin, TX, USA). A significance level of *p* ≤ 0.05 was applied, and statistical tests including one-way ANOVA and Duncan tests were conducted. This study presents the mean and standard deviation (SD) of three measurements.

## 3. Results and Analysis

### 3.1. The Physicochemical Properties of Soil

The application of distiller’s grains biochar, *Lactobacillus plantarum* thallus, the bacterial culture supernatant, and a combined treatment of distiller’s grains biochar and *Lactobacillus plantarum* thallus did not significantly affect the soil pH. However, the soil fertility indicators (nitrogen, phosphorus, potassium, and organic matter) generally showed a pattern of high-dosage treatments > low-dosage treatments > control group after different treatments were applied (Table 2). Specifically, the total nitrogen content in the soil treated with distiller’s grains biochar (T1, T2) and high-dosage biochar–bacteria combinations (T2 + J2) was significantly higher than that of the control group (*p* ≤ 0.05). Conversely, other treatments did not differ significantly from the control group (*p* > 0.05). The changes in soil organic matter content followed a similar pattern to that of total nitrogen. The amount of alkaline nitrogen in the soil was significantly higher in treatments with bacterial thallus and bacterial supernatant compared to the control group (*p* ≤ 0.05). Other treatments did not show any significant differences. Except for the T2 treatment, which did not result in a notable increase in the total phosphorus content (*p* > 0.05), all other treatments demonstrated a notable increase in total phosphorus compared to the control group (*p* ≤ 0.05). Also, the effective phosphorus content in the soil was much higher in the J1, T2, S2, and T2 + J2 treatments compared to the control group (*p* ≤ 0.05). The available potassium content was also significantly greater than that of the control group under treatments T1, T2, S2, and T2 + J2 (*p* ≤ 0.05). However, the soil pH and total potassium content did not significantly differ from the control group across the aforementioned treatments (*p* > 0.05). Overall, the improvement in soil fertility indicators under the high-dosage biochar–bacteria combination treatment (T2 + J2) was superior to that of other treatment groups. Specifically, the total nitrogen, total phosphorus, available phosphorus, available potassium, and organic matter content increased by 46.31%, 43.30%, 16.45%, 47.91%, and 34.70%, respectively, compared to the control group.

### 3.2. Soil Enzyme Activity

**Figure 1:**
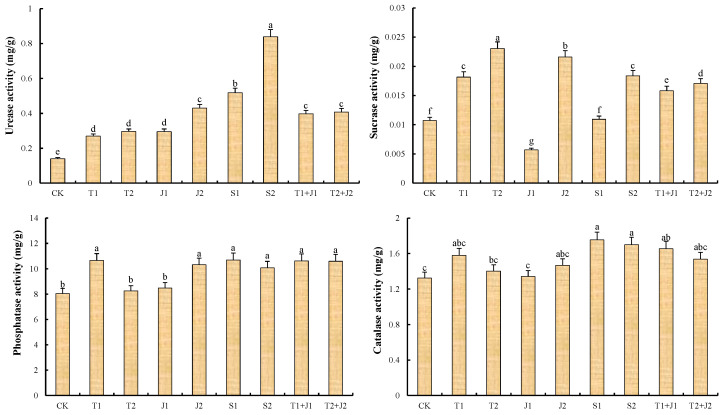
Changes in soil enzyme activity. Different lower cases indicate significant differences among treatments at *p* ≤ 0.05.

Figure 1 illustrates the enzyme activity in the soils under various soil amendment treatments. It can be observed that, compared to the control group CK, the application of distiller’s grains biochar, bacterial thallus, bacterial supernatant, and biochar–bacteria combinations promoted the activity of soil enzymes to a certain extent. Specifically, the soil sucrase activity in the T1, T2, J2, T1 + J1, T2 + J2, and S2 treatments was significantly higher (*p* ≤ 0.05) when compared to the control, showing the following order: T2 > J2 > S2 > T1 > T2 + J2 > T1 + J1, with increases of 53.43%, 50.33%, 41.62%, 40.88%, 32.05%, and 19.03%, respectively. The J1 treatment exhibited significantly lower sucrase activity compared to the control (*p* ≤ 0.05), while the S1 treatment presented no significant difference. The S1 and S2 treatments demonstrated the highest soil urease activity, increasing by 72.93% and 83.29%, respectively, compared to CK. Following these, the J2, T1 + J1, and T2 + J2 treatments showed no significant differences among them, with increases of 67.13%, 65.31%, and 65.61%, respectively. There were no significant differences between the T1, T2, and J1 treatments, but they did increase the urease activity by 47.89%, 52.62%, and 51.97% compared to the CK.

The soil alkaline phosphatase activities in the T1, J2, T1 + J1, T2 + J2, S1, and S2 treatments were higher than those in the CK by 24.32%, 22.09%, 24.55%, 20.24%, 24.83%, and 24.09%, respectively, indicating a significant increase in activity (*p* ≤ 0.05). Additionally, the alkaline phosphatase activities in the T2 and J1 treatments were greater than those in the CK, showing increases of 2.62% and 5.19%, respectively (*p* > 0.05). The soil catalase activities in the T1 + J1, S1, and S2 treatments increased by 19.92%, 20.00%, and 21.03%, respectively, relative to CK. However, the differences between the other treatment groups when amendments were added and the control group were insignificant (*p* > 0.05). Overall, the soil’s catalase activity demonstrated a decreasing trend with increasing amounts of a single material applied, though this trend was not statistically significant. These results show that adding the right amounts of distiller’s grains (biochar, bacterial thallus, and bacterial supernatant) can help break down hydrogen peroxide, protecting organisms from its harmful effects.

### 3.3. The Content of Available Heavy Metals in the Soil

**Figure 2:**
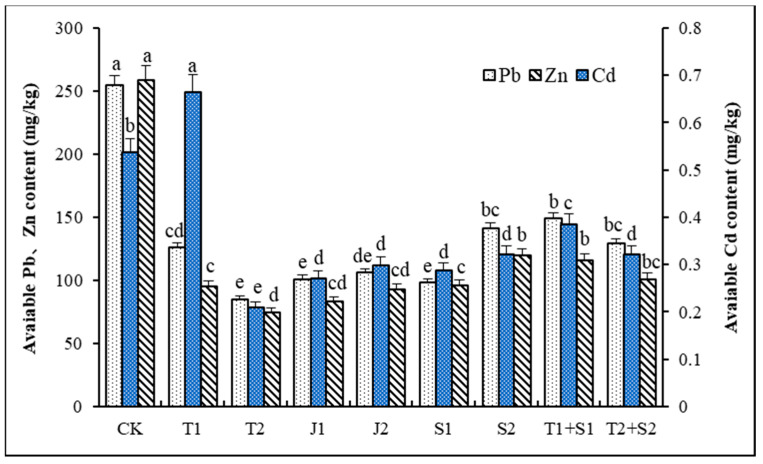
Changes in the available state of heavy metals in soil. Different lower cases indicate significant differences among treatments at *p* ≤ 0.05.

Figure 2 illustrates the contents of available Cd, Pb, and Zn in the soil under different amendment additions. Compared to the control group (CK), the available Cd content in the soil significantly increased under treatment T1 (*p* ≤ 0.05). However, the available Cd levels in the other treatments were significantly lower than those in CK (*p* ≤ 0.05), with the T2 treatment group showing the lowest available Cd content, which decreased by 61.08% compared to CK. There were no significant differences among the available Cd concentrations in the J1, J2, T2 + J2, S1, and S2 treatments, which reduced by 49.53%, 44.32%, 40.22%, 46.55%, and 40.22%, respectively. As observed in Figure 2, the available Pb contents in all treatments were significantly lower than in CK (*p* ≤ 0.05). The most pronounced decreases were observed in the T2, J1, and S1 treatments, with decreases of 60.34%, 62.56%, and 61.40%, respectively. The T1, J2, T1 + J1, T2 + J2, and S2 treatments also exhibited reductions of over 40% compared to CK, with decreases of 50.57%, 58.30%, 41.51%, 49.23%, and 44.57%, respectively. Regarding the available Zn contents, a pattern similar to that observed with Pb was noted, as all treatments recorded significantly lower available Zn levels compared to CK (*p* ≤ 0.05), with reductions ranging from 53.71% to 71.02%. Notably, the T2 treatment demonstrated the most substantial decrease in available Zn, dropping by 71.02% compared to CK.

### 3.4. Plant Growth and Biomass Production

The average plant height and dry weight of Sorghum-sudangrass under various treatments are summarized in Table 3. It is evident that, compared to the control (CK), except for the bacterial thallus addition, the other treatments resulted in varying degrees of plant height increases, ranging from 1.25% to 12.03%. Notably, the plant height was significantly enhanced (*p* ≤ 0.05) with the high dosage of distiller’s grain biochar treatment. When it came to biomass, all of the treatments showed a significant increase in Sorghum-sudangrass biomass. The only treatments that did not show a significant difference were the low dosages of bacterial thallus and supernatant (*p* > 0.05). The specific order of biomass increase was as follows: T2 + J2 > T2 > T1 > S2 > J2 > T1 + J1. These treatments saw increases of 43.15%, 41.88%, 36.19%, 32.78%, 28.29%, and 22.01%, respectively, compared to the CK. In summary, treatments involving distiller’s grain biochar, bacterial thallus and bacterial supernatant all significantly improved the height and dry weight of Sorghum-sudangrass. Furthermore, the enhancement in growth was positively correlated with the increased amounts of the amendments, with the high-dose application of biochar and bacterial agents exhibiting the most effective growth-promoting effects on Sorghum-sudangrass.

### 3.5. Physio-Biochemical Indicators in Plants

#### 3.5.1. Chlorophyll Content

**Figure 3:**
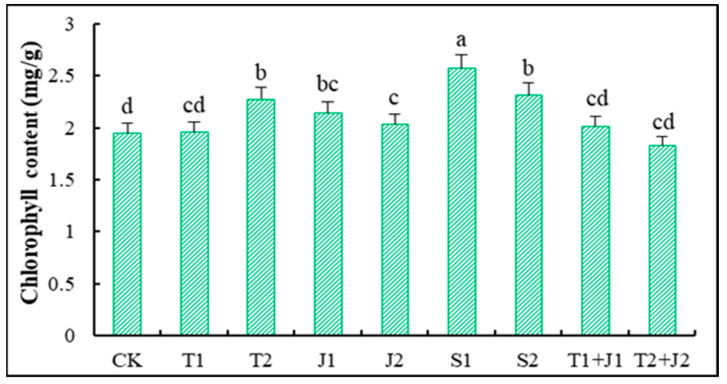
Changes in chlorophyll content of Sorghum-sudangrass. Different lower cases indicate significant differences among treatments at *p* ≤ 0.05.

Figure 3 illustrates the chlorophyll content of the leaves of Sorghum-sudangrass under various treatments. It can be observed that, compared to the control (CK), the chlorophyll content in the various treatments exhibited varying degrees of enhancement. Following the S1 treatment, the chlorophyll content in Sorghum-sudangrass significantly increased (*p* ≤ 0.05) relative to the control, with the maximum increase being 29.11%. The T2, J1, and S2 treatments also significantly boosted the chlorophyll content (*p* ≤ 0.05), with increases of 19.80%, 14.87%, and 21.05%, respectively; however, there were no significant differences among these three treatments The amount of chlorophyll also went up after the T1, T1 + J1, and T2 + J2 treatments compared to the control (6.59%, 9.21%, and 6.06%, respectively), but these differences were not statistically significant (*p* > 0.05). Overall, the results indicate that the application of the aforementioned remediation materials under heavy metal stress conditions is beneficial for alleviating heavy metal stress and promoting the synthesis of chlorophyll in Sorghum-sudangrass.

#### 3.5.2. Soluble Sugar Content

**Figure 4:**
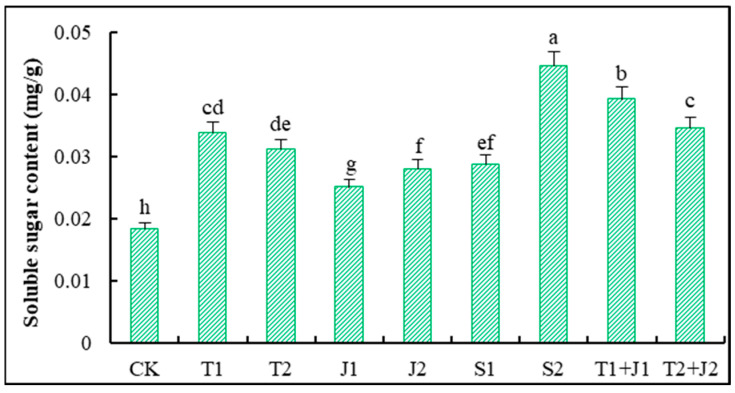
Changes in soluble sugar content of Sorghum-sudangrass. Different lower cases indicate significant differences among treatments at *p* ≤ 0.05.

Soluble sugars are critical indicators of the carbon nutritional status of plants and the quality characteristics of agricultural products [43]. Figure 4 shows that the amount of soluble sugar in the aerial parts of Sorghum-sudangrass rose significantly after the amendments were added compared to the control (*p* ≤ 0.05). Among the treatments, the S2, T1 + J1, and T2 + J2 groups exhibited the highest increases in soluble sugar content, with respective increases of 53.14%, 46.93%, and 58.81% compared to CK. Other treatments also demonstrated significant enhancements in the soluble sugar content compared to CK (*p* ≤ 0.05), with increases ranging from 26.49% to 45.67%.

#### 3.5.3. Soluble Protein Content

**Figure 5:**
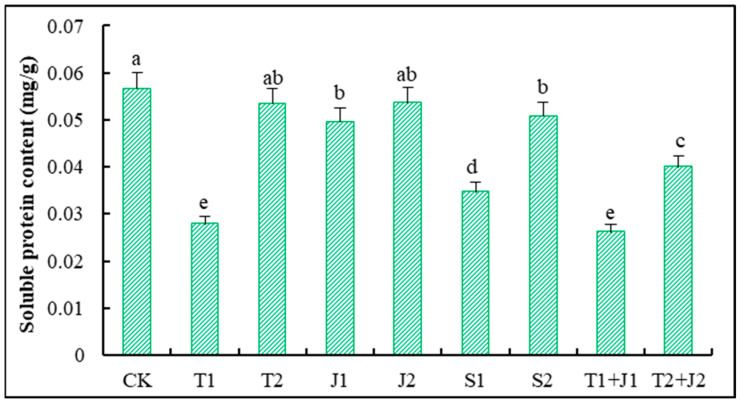
Changes in soluble protein content of Sorghum-sudangrass. Different lower cases indicate significant differences among treatments at *p* ≤ 0.05.

Soluble proteins serve as vital nutrients and osmoregulatory substances for plants. Under conditions of heavy metal stress, plants can modulate their internal osmotic potential by altering the levels of soluble proteins, which helps mitigate the adverse effects of such stressors on plant health [43]. As illustrated in Figure 5, the addition of various amendments led to varying reductions in the soluble protein content of the aerial parts of Sorghum-sudangrass grown in soils contaminated with heavy metals. The amounts of soluble protein in the T1, T1 + J1, T2 + J2, and S1 treatments were significantly lower than the control (*p* ≤ 0.05), dropping by 50.84%, 53.80%, 29.42%, and 38.76%, respectively. Conversely, the CK, T2, J1, J2, and S1 treatments showed no significant differences in the soluble protein content (*p* > 0.05).

#### 3.5.4. Activity of Superoxide Dismutase (SOD)

**Figure 6:**
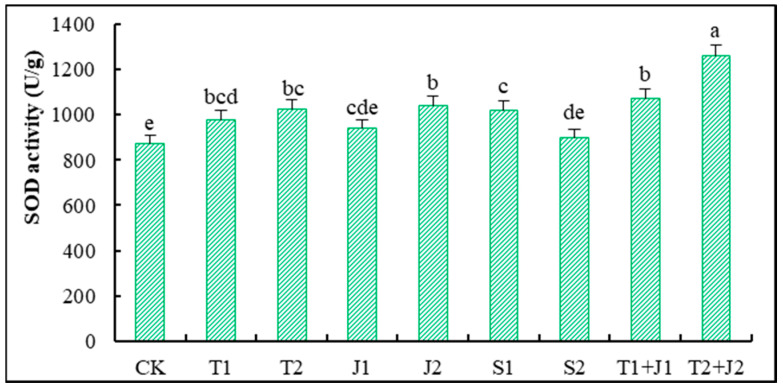
Changes in superoxide dismutase activity in Sorghum-sudangrass. Different lower cases indicate significant differences among treatments at *p* ≤ 0.05.

Superoxide dismutase (SOD) is an essential antioxidant enzyme that helps combat oxidative stress in living organisms [43]. As shown in Figure 6, adding amendments increased the SOD activity in the aerial parts of Sorghum-sudangrass to varying degrees compared to the control (CK). Except for the J1 and S2 treatments, the other treatments significantly enhanced the SOD activity (*p* ≤ 0.05). One of the most impressive results was when a high dose of biochar was mixed with a bacterial agent (T2 + J2). This mixture increased the SOD activity by 30.66% compared to the control. These findings indicate that the aforementioned remediation materials can effectively mitigate heavy metal toxicity by enhancing the SOD activity in Sorghum-sudangrass. Heavy metal toxicity is reduced most when biochar and bacterial agents are used in large amounts. This is because they greatly increase the SOD activity.

#### 3.5.5. Proline Content

**Figure 7:**
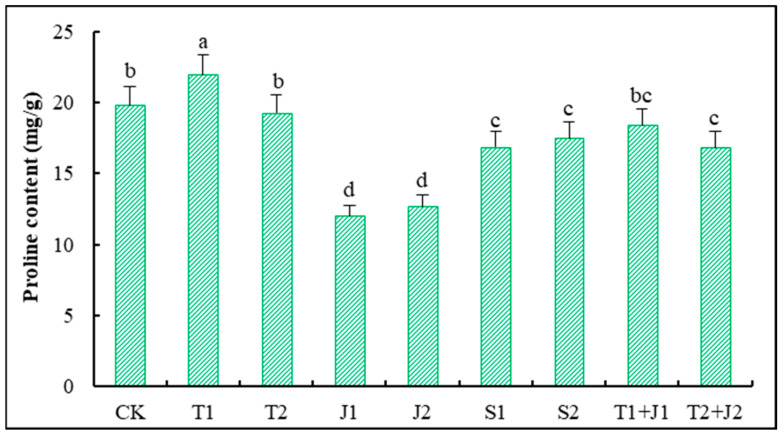
Changes in proline content in Sorghum-sudangrass. Different lower cases indicate significant differences among treatments at *p* ≤ 0.05.

The proline content in plants serves as a biochemical indicator of stress-related damage [43]. Figure 7 presents the proline levels in the aerial parts of Sorghum-sudangrass subjected to different treatments. Notably, the T1 treatment significantly increased the proline content compared to the control group (*p* ≤ 0.05). However, there was no significant difference in the proline levels between CK, T2, and T1 + J1 (*p* > 0.05). In contrast, under the treatments involving bacterial thallus, bacterial supernatants, and a high dosage of the biochar–microbe combination, the proline content in Sorghum-sudangrass significantly decreased (*p* ≤ 0.05), with reductions of 39.54%, 36.11%, 15.08%, 14.93%, and 11.80%, respectively, relative to CK. This decrease in proline content can effectively reduce cellular osmotic pressure, thereby enhancing the plant’s ability to cope with heavy metal stress.

#### 3.5.6. Malondialdehyde (MDA) Level

**Figure 8:**
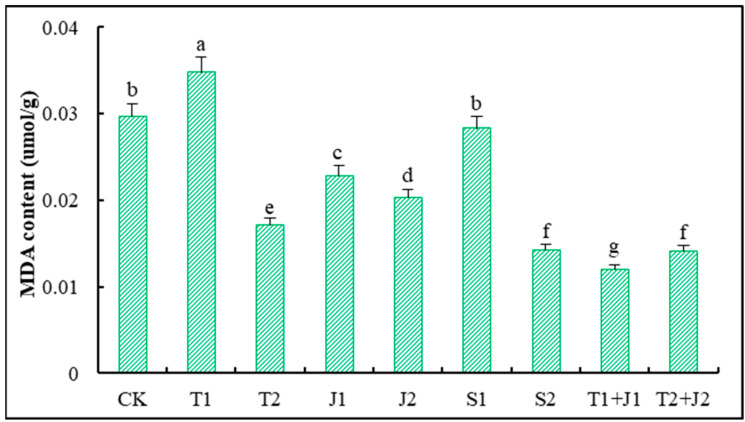
Changes in malondialdehyde content in Sorghum-sudangrass. Different lower cases indicate significant differences among treatments at *p* ≤ 0.05.

The level of (MDA) serves as an indicator of lipid peroxidation in plant cell membranes [43]. Figure 8 shows the MDA levels in the tops of Sorghum-sudangrass plants after different treatments. The T1 treatment significantly raised MDA levels compared to the CK treatment (*p* ≤ 0.05). Conversely, treatments T2, J1, J2, T1 + J1, T2 + J2, and S2 effectively reduced the MDA levels (*p* ≤ 0.05), with decreases of 42.53%, 23.13%, 31.65%, 59.93%, 52.53%, and 52.19%, respectively, compared to the control. The application of biochar and bacterial agents significantly lowered the MDA content in Sorghum-sudangrass, with reductions exceeding 50%. This decrease effectively mitigates the oxidative damage induced by heavy metal stress, indicating a strong potential for plant recovery.

### 3.6. The Accumulation of Heavy Metals in Plants

**Figure 9:**
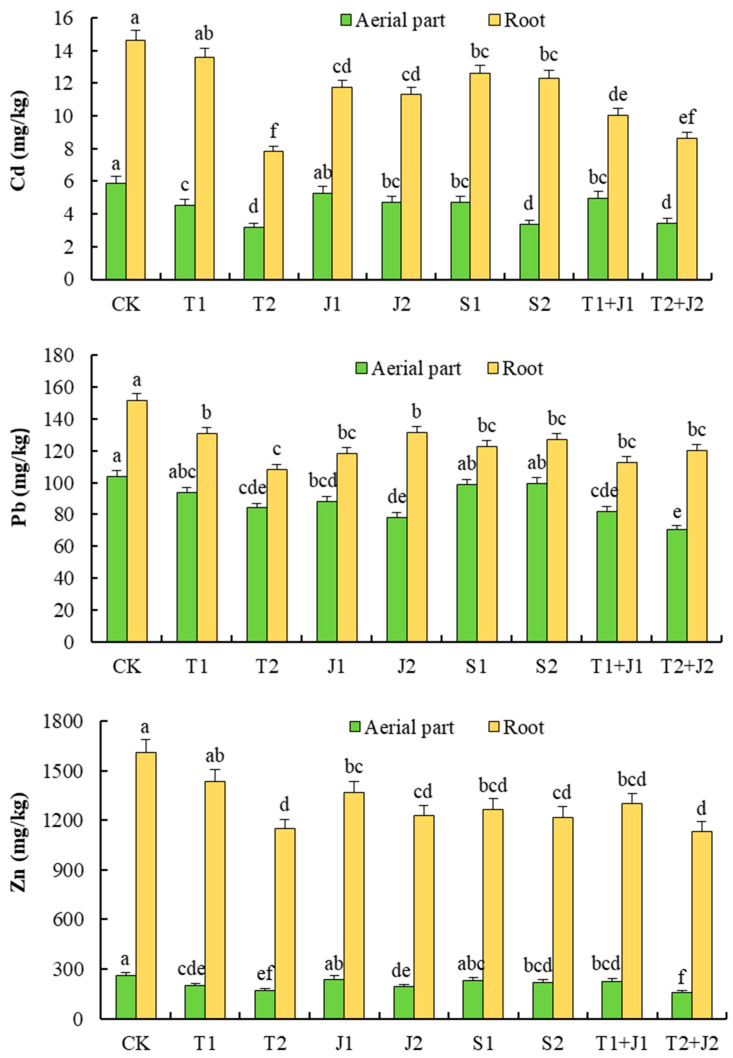
Contents of Cd, Pb, and Zn in the aerial parts and roots of Sorghum-sudangrass under different treatments. Different lower cases indicate significant differences among treatments at *p* ≤ 0.05. The results for the Pb content in aerial parts are the data multiplied by 20.

The content of Cd, Pb, and Zn in the aerial parts and roots of Sorghum-sudangrass following the addition of various materials is presented in Figure 9. It is evident that, compared to the control group (CK), the standalone and combined application of distiller’s grains biochar, *Lactobacillus plantarum* thallus, and the bacterial supernatant all effectively reduced the levels of Cd, Pb, and Zn in the aerial parts and roots of the test plants to varying extents. In terms of the Cd content (Figure 9), except for the J1 treatment, all treatments demonstrated significant reductions in the aerial parts compared to CK (*p* ≤ 0.05). Particularly effective were the T2, T2 + J2, and S2 treatments, which achieved reductions exceeding 40%, with decreases of 45.79%, 40.93%, and 42.69%, respectively. The T2 and T2 + J2 treatments showed the most significant reductions (*p* ≤ 0.05), achieving decreases of 46.67% and 41.09%, respectively, when compared to CK.

For the Pb levels (Figure 9), treatments T1, S1, and S2 saw small but not significant reductions in the aerial parts compared to CK (*p* > 0.05). Other treatments, on the other hand, saw significant reductions (*p* ≤ 0.05), with T2 + J2 being the most effective, lowering the Pb levels by 31.84% compared to CK. All of the treatments showed significant decreases in Pb levels compared to CK in the roots (*p* ≤ 0.05), but T2 was the most effective, lowering the levels by 28.44%. T1+ J1 and T2 + J2 were next, lowering the levels by 25.44% and 21.79%, respectively. As shown in Figure 9, all treatments significantly decreased the plants’ zinc levels. The only ones that did not make a big difference were the J1 and S1 treatments, which decreased the zinc content in the plant’s leaves, and the T1 treatment, which decreased the zinc content in the plant’s roots. Notably, the T2 + S2 treatment resulted in the most pronounced reduction, with the zinc content in the aboveground and root parts decreasing by 40.25% and 29.62%, respectively, compared to the control.

## 4. Discussion

The results of this study on soil physicochemical indicators reveal that the treatments involving distiller’s grain biochar, including both single applications and applications combined with bacterial thallus, significantly increased soil-related fertility levels, with the combined application proving more effective than the single application. Biochar’s larger specific surface area, well-developed pore structure, and diverse surface functional groups all contribute to its enhanced ability to retain moisture and nutrient elements in the soil [22,26]. As a result, biochar effectively decreases nutrient leak losses and improves soil fertility. Furthermore, the adsorption properties of biochar’s surface, along with its pore protection and mineral nutrients, provide an excellent environment for the habitation and proliferation of microorganisms [44], thereby increasing the abundance and diversity of soil microorganisms. Soil microorganisms are capable of decomposing and transforming soil organic matter, releasing nutrient elements, and boosting soil nutrient content. Additionally, they can degrade harmful substances remaining in the soil, leading to an effective improvement in the soil’s physicochemical properties [45,46].

It was found that adding biochar made from distiller’s grains, *Lactobacillus plantarum thallus,* and bacterial culture supernatant increased the activities of soil enzymes like sucrose, urease, alkaline phosphatase, and catalase compared to the control group. For soil sucrose and urease, higher enzyme activities were observed in the high-dosage treatments compared to the low-dosage treatments when the same amendment was added. Conversely, the activities of soil alkaline phosphatase and catalase demonstrated a decreasing trend with increasing amounts of the amendments. This finding suggests that the appropriate application of the aforementioned remediation materials can effectively enhance soil enzyme activity; however, excessively high application amounts may exert inhibitory effects on some enzymes. This could be attributed to the large specific surface area and porous structure of biochar, which enable it to effectively adsorb substrates for enzymatic reactions and enhance soil enzyme activity [46,47]. Additionally, biochar can provide soil microorganisms with a suitable living environment and essential nutrients for growth, such as carbon and nitrogen sources, which may alter the composition and abundance of soil microbial communities [48,49]. Soil microorganisms play a crucial role in secreting and releasing soil enzymes, significantly impacting material cycling and energy flow within the soil, and thus affecting overall soil enzyme activity. However, the response of enzymatic activity to biochar depends on the feedstock material and the incubation time. Teutscherova et al.’s research revealed that biochar application enhanced the activity of dehydrogenase and urease in Acrisol, while it either decreased or had no effect on the activity of all other enzymes [50]. Wojewódzki et al.’s study also demonstrated that biochar application inhibits soil enzyme activity 60 days later [51]. This is attributed to the excessive amounts of biochar that may adsorb enzyme molecules, creating a protective barrier that inhibits access to the active sites of these enzymes, thus hindering enzymatic reactions [52]. Also, biochar might release harmful chemicals like polycyclic aromatic hydrocarbons, dioxins, and phthalates. These chemicals could lower the activity of microbes and enzymes in the soil when a lot of biochar is applied [53].

In this study, the bioavailable Cd levels in all but the T1 treatment were significantly lower than those in CK (*p* ≤ 0.05), with reductions of more than 40%. Similarly, the bioavailable levels of Pb and Zn in all treatments were significantly lower than in CK (*p* ≤ 0.05), with reductions of over 40% for Pb and over 50% for Zn. These indicate that the addition of the amendments effectively transformed the unstable forms of Cd, Pb, and Zn in the soil into stable forms, demonstrating excellent immobilization effects and greatly reducing the bioavailability of these heavy metals. Overall, the T2 treatment achieved the most pronounced reductions, with the bioavailable Cd, Pb, and Zn levels decreasing by 61.08%, 60.34%, and 71.02% compared to CK, respectively, thereby substantially lowering the bioavailability of heavy metals. The absorption and accumulation of heavy metals by plants are influenced by numerous factors, including the soil’s pH, its fertility, the availability of heavy metals, and microbial community structure [54]. The high pH, unique surface structure, and abundant functional groups of biochar enable it to engage in complex reactions with heavy metal ions, such as ion exchange, electrostatic adsorption, surface complexation, or co-precipitation [23,55]. These properties give biochar a strong adsorption capacity for soil heavy metals, effectively reducing their mobility and bioavailability. Biochar exhibits strong adsorption for heavy metals and can also serve as an effective carrier for microorganisms, which contributes to the recovery of microbial diversity and functionality and subsequently promotes the microbial transformation of certain bioavailable heavy metals [56]. Additionally, active groups on the surface of microbial cells, such as amide, hydroxyl, and carbonyl groups, can effectively adsorb heavy metal ions, thereby reducing their bioavailability and alleviating the stress they impose on plants [57]. In the present study, the use of distiller’s grains biochar, *Lactobacillus plantarum* thallus, and the bacterial culture supernatant as remediation materials significantly reduced the bioavailable contents of heavy metals Cd, Pb, and Zn in the soil, demonstrating great potential for remediating metal-contaminated soils.

The distiller’s grains biochar and *Lactobacillus plantarum* agents not only reduce the bioavailable content of heavy metals in the soil and improve the soil’s physicochemical properties, but they also promote plant growth, increasing the height and fresh weight of the hybridized Sorghum and sudangras. Furthermore, the growth enhancement increases with the amount of added material. This may be explained by the fact that appropriate amounts of biochar can retain moisture and nutrients and improve soil’s physicochemical properties, as well as promote the growth and reproduction of rhizosphere microorganisms. As key decomposers in the soil, these microorganisms accelerate the breakdown of organic matter and minerals, thereby enhancing plant growth and the soil’s physiological metabolic activities. Furthermore, the incorporation of these materials not only mitigates the impact of heavy metals but also fosters the growth of plants.

Plants’ levels of chlorophyll, soluble sugars, soluble proteins, superoxide dismutase (SOD), proline, and malondialdehyde (MDA) change when they are stressed from the outside. These changes can serve as indicators for assessing the extent of stress damage and plant adaptability [43]. The changes in chlorophyll content are usually associated with the physiological activity, environmental adaptability, and stress resistance of plants. Plants tend to decrease their chlorophyll levels when exposed to external environmental stress, and their capacity to withstand stress influences the extent of this decline; those with stronger stress resistance experience a lower reduction in chlorophyll [58]. The results of this study show that adding the materials made the chlorophyll content in Sorghum-sudangrass much higher than it was with CK. This was achieved by reducing the harmful effects of heavy metal stress on chlorophyll synthesis and increasing its production, which is good for photosynthesis. The abundant nutrients in biochar and microbial cultures, which are essential for photosynthesis, may contribute to this enhancement. Additionally, the adsorption properties of biochar reduce nutrient loss, thereby regulating nutrient cycling in the soil and maintaining soil fertility. This, in turn, provides ample nutrient resources for chlorophyll synthesis; within a certain range, the greater availability of nutrient elements correlates with faster chlorophyll synthesis and a higher photosynthetic rate.

The soluble sugar content serves as an important indicator of the carbon nutritional status, product quality, and osmoregulatory substances in plants [43,59]. The results of this experiment reveal that the application of distiller’s grains, biochar, *Lactobacillus plantarum* thallus, and the bacterial culture supernatant significantly elevated the soluble sugar content in Sorghum-sudangrass, compared to CK. This increase can be attributed to the fact that biochar is rich in stable carbon elements that decompose slowly, while abundant soil microorganisms accelerate its decomposition, thereby raising the carbon content and supplying sufficient metabolic substrates for the synthesis and transformation of soluble sugars, which ultimately enhances carbon metabolic products. Soluble proteins play a critical role as osmoregulatory substances and nutrients, protecting cellular life and biological membranes [59]. In heavy-metal-contaminated soils, the addition of the aforementioned materials resulted in varying reductions in the soluble protein content in Sorghum-sudangrass. The most pronounced reductions were observed with low dosages of distiller’s grains biochar, low dosages of microbial supernatants, and combined treatments. There were notable differences in the soluble protein content among the various treatments, with higher soluble protein levels found in the high-dosage treatments compared to their low-dosage counterparts. This variability may be related to the intrinsic characteristics of the amendments, their carbon-to-nitrogen ratios, and the quantities applied.

The alterations in the superoxide dismutase (SOD) activity within plants serve as a crucial response mechanism to stress conditions, reflecting changes in plant metabolism and stress resistance under adverse circumstances [43,60]. In this study, the application of distiller’s grains biochar, Lactobacillus plantarum thallus, and the bacterial culture supernatant significantly increased the SOD activity in Sorghum-sudangrass compared to CK, with the high-dosage biochar–bacteria combined treatment showing the most pronounced effect. This suggests that these materials can mitigate heavy metal toxicity by increasing the SOD activity, with the combined treatment at a high amount demonstrating the greatest efficacy in alleviating such toxicity. Proline is an essential osmotic regulator that scavenges reactive oxygen species within plants, mitigates lipid peroxidation, and plays a critical role in regulating cellular osmotic balance [43,61]. The results of this study indicate that, except for the low-dosage distiller’s grain biochar treatment, all other treatments led to varying reductions in the proline levels in Sorghum-sudangrass compared to the control group. This reduction effectively decreased cell osmotic pressure and enhanced the ability of Sorghum-sudangrass to withstand heavy metal stress.

Malondialdehyde (MDA), a final product of membrane lipid peroxidation under stress, serves as an indicator of the extent of damage to plant lipid membranes [62]. Except for the low-dosage distiller’s grain biochar treatment, this study found that all other treatments significantly reduced the MDA levels in Sorghum-sudangrass compared to the control group, suggesting that they can effectively lessen the damage heavy metals inflict on the plasma membranes of Sorghum-sudangrass. This response can be attributed to the generation of large amounts of reactive oxygen species under heavy metal stress, which leads to membrane lipid peroxidation and cell membrane damage. Biochar can enhance soil porosity, decrease soil’s bulk density, increase its organic matter, and improve its fertility and environmental conditions, thereby fostering greater microbial diversity and community structure. This, in turn, aids microorganisms in transporting heavy metal ions into cells and converting them into more stable forms, alleviating heavy metal stress.

## 5. Conclusions

The plant height, biomass, chlorophyll content, soluble sugar levels, and superoxide dismutase activity of Sorghum-sudangrass were all improved to varying degrees by the standalone and combined application of distiller’s grains biochar, *Lactobacillus plantarum* thallus, and the bacterial supernatant. Further, these treatments reduced the levels of proline and malondialdehyde in the test plants, indicating that the addition of these materials can effectively promote plant growth and alleviate the damage caused by heavy metal stress. Furthermore, the application of the aforementioned remediation materials can enhance soil fertility indicators such as nitrogen, phosphorus, potassium, and organic matter. It can also boost the activity of soil enzymes, including urease, sucrase, phosphatase, and peroxidase. Additionally, it can decrease the content of available heavy metals (Cd, Pb, and Zn) in the soil and inhibit the uptake of heavy metals by both the aerial and root parts of the plants, contributing to improved growth conditions. The combined application of high doses of distiller’s grain biochar and *Lactobacillus plantarum* thallus can achieve optimal soil improvement and heavy metal passivation effects, resulting in the best plant growth outcomes.

## Figures and Tables

**Table 1:** Amount of amendments added.

Lables	Treatments
CK	Control group
T1	2.2% distiller’s grains biochar
T2	4.4% distiller’s grains biochar
J1	0.0125% *Lactobacillus plantarum* thallus
J2	0.025% *Lactobacillus plantarum* thallus
S1	0.055 mL·g^−1^ *Lactobacillus plantarum* supernatant
S2	0.11 mL·g^−1^ *Lactobacillus plantarum* supernatant
T1 + J1	2.2% distiller’s grains biochar + 0.0125% *Lactobacillus plantarum* thallus
T2 + J2	4.4% distiller’s grains biochar + 0.025% *Lactobacillus plantarum* thallus

**Table 2:** Physical and chemical properties of soil in different application treatments. Different lower cases indicate significant differences among treatments at *p* ≤ 0.05.

Treatment	pH	Total Nitrogen (g/kg)	Alkaline Nitrogen (mg/kg)	Total Phosphorus (g/kg)	Available Phosphorus (mg/kg)	Total Potassium (g/kg)	Available Potassium (mg/kg)	Organic Matter (g/kg)
CK	7.63 ± 0.38 a	1.16 ± 0.06 c	85.18 ± 4.26 ef	0.43 ± 0.02 e	23.84 ± 1.19 de	6.95 ± 0.35 a	112.58 ± 5.63 d	42.53 ± 2.13 b
T1	7.61 ± 0.38 a	1.81 ± 0.09 b	89.18 ± 4.46 def	0.52 ± 0.03 de	21.02 ± 1.05 e	7.6 ± 0.38 a	137.1 ± 6.86 c	60.26 ± 3.01 a
T2	7.73 ± 0.39 a	2.36 ± 0.12 a	80.81 ± 4.04 f	0.91 ± 0.05 a	35.45 ± 1.77 a	7.18 ± 0.36 a	181 ± 9.05 b	64.47 ± 3.22 a
J1	7.62 ± 0.38 a	1.29 ± 0.06 c	105.93 ± 5.3 bc	0.61 ± 0.03 cd	28.07 ± 1.4 bc	6.86 ± 0.34 a	112.82 ± 5.64 d	47.72 ± 2.39 b
J2	7.74 ± 0.39 a	1.35 ± 0.07 c	127.69 ± 6.38 a	0.66 ± 0.03 bc	25.92 ± 1.3 bcd	7.07 ± 0.35 a	131.44 ± 6.57 cd	48.3 ± 2.42 b
S1	7.71 ± 0.39 a	1.29 ± 0.06 c	101.11 ± 5.06 bcd	0.64 ± 0.03 c	25.38 ± 1.27 bcd	7.15 ± 0.36 a	134.77 ± 6.74 cd	45.46 ± 2.27 b
S2	7.63 ± 0.38 a	1.24 ± 0.06 c	108.73 ± 5.44 b	0.88 ± 0.04 a	28.92 ± 1.45 b	7.08 ± 0.35 a	143.93 ± 7.2 c	46.72 ± 2.34 b
T1 + J1	7.71 ± 0.39 a	1.37 ± 0.07 c	91.68 ± 4.58 cdef	0.56 ± 0.03 cd	24.4 ± 1.22 cde	7.03 ± 0.35 a	133.78 ± 6.69 cd	49.02 ± 2.45 b
T2 + J2	7.77 ± 0.39 a	2.16 ± 0.11 a	96.5 ± 4.82 bcde	0.75 ± 0.04 b	28.54 ± 1.43 bc	6.55 ± 0.33 a	216.12 ± 10.81 a	65.14 ± 3.26 a

**Table 3:** Effects of different treatments on the growth of Sorghum-sudangrass. Different lower cases indicate significant differences among treatments at *p* ≤ 0.05.

Treatments	Average Plant Height (cm)	Dry Weight (g/plant)
CK	79.00 ± 3.95 bcd	3.65 ± 0.18 e
T1	82.40 ± 4.12 bc	5.72 ± 0.29 b
T2	89.80 ± 4.49 a	6.28 ± 0.31 a
J1	73.60 ± 3.68 d	3.43 ± 0.17 e
J2	74.40 ± 3.72 d	5.09 ± 0.25 cd
S1	76.10 ± 3.80 cd	3.72 ± 0.24 e
S2	80.00 ± 4.00 bcd	5.43 ± 0.27 bc
T1 + J1	82.10 ± 4.10 bc	4.68 ± 0.23 d
T2 + J2	86.60 ± 4.33 ab	6.42 ± 0.32 a

## Data Availability

The original contributions presented in this study are included in the article. Further inquiries can be directed to the corresponding authors.
